# The Impact of Risk Score Use in Predicting Serious Adverse Events During Cardiac Catheterization Procedures in Pediatric Patients

**DOI:** 10.3390/jcm14196919

**Published:** 2025-09-30

**Authors:** Muhammet Hamza Halil Toprak, Hatice Dilek Özcanoğlu, İbrahim Akkoç, Kahraman Yakut, Ali Nazım Güzelbağ, Abdullah Erdem, İbrahim Cansaran Tanıdır, Erkut Öztürk

**Affiliations:** 1 Department of Pediatric Cardiology, Istanbul Saglik Bilimleri University Basaksehir Cam and Sakura Hospital, Istanbul 34480, Turkey; kahramanyakut@gmail.com (K.Y.); anguzelbag@gmail.com (A.N.G.); drabdullaherdem@hotmail.com (A.E.); cansaran@yahoo.com (İ.C.T.); erkut_ozturk@yahoo.com (E.Ö.); 2 Department of Anesthesiology and Reanimation, Istanbul Saglik Bilimleri University Basaksehir Cam and Sakura Hospital, Istanbul 34480, Turkey; dilekmersin@hotmail.com (H.D.Ö.); ibrakkoc@gmail.com (İ.A.)

**Keywords:** adverse event, cardiac catheterization, children, congenital heart disease, risk score

## Abstract

**Background**: Cardiac catheterization may be required in the management of congenital heart diseases. The use of risk scoring or grading systems in these procedures can assist in planning the intervention and predicting potential complications. This study aimed to evaluate the use of risk scores in grading cardiac catheterization procedures in pediatric patients and to investigate their predictive value for serious adverse events (SAEs). **Material and Methods**: A total of 700 pediatric patients (350 male; median age 1 year [IQR 6 months–2 years]) who underwent cardiac catheterization in our catheterization laboratory between 1 January 2023 and 1 January 2025 were retrospectively analyzed. Demographic and clinical data of the patients, including procedure duration, anesthesia management, Catheterization Risk in Pediatrics Score (CRISP), and serious adverse events related to the procedure, were recorded. The results were analyzed statistically. **Results**: In total, 50% of the patients were male (*n* = 350), and 58% (*n* = 406) had single-ventricle physiology. Interventional procedures were performed in 72% of the cases. The median CRISP score was 8 (IQR 6–10). SAEs occurred in 7.7% of the patients (*n* = 54), most of which were arrhythmia-related. The incidence of SAEs was analyzed according to CRISP score categories. The rates of SAEs in patients with CRISP Categories 1 through 5 were 2.9%, 4.3%, 11%, 17.3%, and 41%, respectively. As the CRISP score and category increased, the incidence of SAEs also increased [area under the curve of 0.84 (95% confidence interval, 0.76–0.92; *p* < 0.05)]. **Conclusions**: CRISP may serve as an effective benchmarking and risk classification tool in pediatric cardiac catheterization procedures and can predict SAE occurrence. Therefore, it may have a positive impact on patient care by assisting in the planning of pre- and post-catheterization care.

## 1. Introduction

Congenital heart disease (CHD) is a relatively common condition, occurring in four to ten cases per 1000 live births, making it the most frequent birth defect worldwide. The global prevalence has been steadily increasing over recent decades, partly due to improved diagnostic techniques and enhanced survival rates. Current epidemiological data suggest that approximately 1.35 million children are born with CHD annually worldwide, representing a significant healthcare burden. The incidence varies geographically due to genetic, environmental, and socioeconomic factors, including maternal age, diabetes, and access to prenatal care.

CHDs are a group of disorders with a wide spectrum of pathologies and subtypes, and treatment approaches can vary significantly. Accurate and timely diagnosis and treatment are crucial to improving survival outcomes for affected patients [1]. The complexity of these lesions ranges from simple defects such as isolated atrial or ventricular septal defects to highly complex anomalies including single-ventricle physiology or transposition of the great arteries. Classification systems help standardize lesion complexity assessment.

Beyond the immediate clinical implications, CHDs constitute a major cause of morbidity and mortality in the pediatric population and are associated with considerable long-term healthcare costs. Economic analyses have demonstrated that the lifetime healthcare expenditure for individuals with CHD can exceed several hundred thousand dollars per patient, depending on the complexity of the lesion. The societal burden extends beyond direct medical costs to include indirect costs such as lost productivity and psychological support for families. Furthermore, advances in medical care have led to a growing population of adults with CHD, estimated to exceed one million individuals in the United States alone.

Electrocardiography (ECG) and echocardiography (ECHO) remain the cornerstone diagnostic tools for evaluating heart disease in children due to their non-invasive nature and high diagnostic accuracy. However, in cases where these methods are insufficient to provide adequate anatomical or hemodynamic information, additional imaging modalities may be required. Advanced imaging techniques such as cardiac magnetic resonance imaging (MRI), computed tomography (CT), and three-dimensional echocardiography have expanded diagnostic capabilities. Nevertheless, invasive cardiac catheterization remains the gold standard for hemodynamic assessment and is indispensable for many interventional procedures.

Pediatric cardiac catheterization is typically performed for the diagnosis and interventional treatment of congenital heart diseases or hemodynamic problems [2,3]. The evolution from a purely diagnostic procedure to a predominantly interventional modality reflects advances in catheter technology and device development. Modern catheterization laboratories are equipped with sophisticated biplane fluoroscopy systems and an extensive array of therapeutic devices including balloons, stents, occluders, and valves.

However, cardiac catheterization carries a range of potential complications, which can vary significantly based on patient characteristics and procedural complexity. Historical data from the early era of pediatric catheterization reported complication rates as high as 10–15%, with mortality rates exceeding 1%. Advances in catheterization techniques, equipment miniaturization, and improved procedural protocols have led to substantially improved complication rates. Contemporary series from high-volume centers report overall complication rates of 2–8%, with mortality rates typically below 0.1%.

Previous studies have identified several risk factors that are associated with complications during cardiac catheterization procedures [4,5]. Patient-related variables include young age (particularly neonates), low body weight (especially patients under 5 kg), the presence of cyanosis, significant ventricular dysfunction, and associated extracardiac anomalies. Procedure-related risk factors encompass the complexity of the planned intervention, duration of the procedure, use of general anesthesia versus sedation, and operator experience. Institutional factors such as case volume and availability of on-site cardiac surgery also influence complication rates.

To address the need for a standardized method of risk stratification, the Catheterization Risk in Pediatrics Score (CRISP) was developed through a collaborative effort involving multiple high-volume pediatric cardiac centers. The CRISP score is an empirically based risk assessment tool created specifically for pediatric cardiac catheterization, utilizing real-world data from thousands of procedures. It was developed using comprehensive data from the Congenital Cardiovascular Interventional Study Consortium (CCISC) registry and has since been refined to utilize only pre-procedure data [6]. This characteristic makes it highly valuable, as it allows for early prediction of procedural risk before the intervention begins.

CRISP evaluates 8 key variables through an 11-component scoring system—namely, age, body weight, need for inotropic support, presence of systemic illness or organ failure, physiological category, pre-catheterization diagnosis, procedure risk category, and procedure type—via a web-based calculator. Each variable is weighted to reflect its independent contribution to procedural risk, thereby enabling clinicians to stratify patients into discrete categories of predicted risk. CRISP is widely used to predict the likelihood of any serious adverse event (SAE), and its implementation has been associated with improved pre-procedural planning, resource allocation, and patient counseling in many high-volume pediatric cardiac centers [3,6].

In this study, we aimed to assess the use of CRISP scoring in pediatric cardiac catheterization procedures and to investigate its potential predictive value for the occurrence of serious adverse events (SAEs). Additionally, we sought to evaluate the score’s performance in a population with a high prevalence of single-ventricle physiology and complex interventional procedures.

## 2. Materials and Methods

A total of 700 pediatric patients (350 males; median age 1 year [IQR 6 months–2 years]) who underwent cardiac catheterization in our catheterization laboratory between 1 January 2023, and 1 January 2025, were retrospectively analyzed. Patients born prematurely (*n* = 15), those over the age of 18 (*n* = 24), patients who underwent angiography for electrophysiological studies (*n* = 120), those with inaccessible medical records(*n* = 18) were excluded from this study.

The following data were obtained from patients’ anaesthesia follow-up forms: demographic data; American Society of Anaesthesiologists (ASA) classification; CRISP score; type of cardiac catheterisation; anaesthesia management; and procedure duration (defined as the time from catheter insertion to removal). Data regarding SAEs were also obtained. These parameters were selected because they provide comprehensive information on both patient-related and procedure-related risk factors, thereby allowing for a detailed evaluation of outcomes. This study was planned in accordance with the ethical principles outlined in the Declaration of Helsinki, which ensures respect for patient rights, confidentiality, and the responsible conduct of research. The data presented in this study were collected retrospectively from medical records and reviewed systematically to minimize bias and maintain data integrity. Approval was obtained from Ethics Committee of Istanbul Çam and Sakura City Hospital, University of Health Sciences of Turkey (protocol code: protocol code: KAEK/29.01.2025.31, date: 06.02.2025), and all procedures were carried out under institutional guidelines for clinical research. Given the retrospective nature of this study, informed consent was waived, yet strict attention was paid to protecting patient anonymity and ensuring that no identifiable information was disclosed. This rigorous methodological and ethical framework enhances the validity, reproducibility, and clinical relevance of the study findings.

### 2.1. Anesthesia Approach and Procedure Characteristics

The procedures were performed by two operators (each with at least five years of experience) and three different teams. All procedures were performed under sterile conditions in a pediatric angiography suite using the Philips Biplane Azurion 7 B12/12 (Philips Medical Systems International B.V., Best, The Netherlands) image-guided therapy system. The catheterization laboratory was equipped with advanced hemodynamic monitoring capabilities, emergency resuscitation equipment, and immediate access to cardiac surgical backup when indicated. Procedures were conducted under general anesthesia (with laryngeal mask or intubation) or sedoanalgesia, using appropriately sized sheaths and catheters based on the planned intervention. A percutaneous technique was used for all interventions. Right heart catheterization was performed via the femoral vein, and left heart catheterization via the femoral artery. Depending on the technical requirements of the procedure, alternative access routes such as axillary, carotid, or umbilical artery/vein were occasionally utilized. Vascular access site selection was individualized based on patient anatomy, procedure complexity, and operator preference to minimize complications. In all cases, vascular access was guided by ultrasonography to enhance precision and reduce vascular trauma.

All patients were continuously monitored using electrocardiography (ECG), as well as monitoring of their peripheral oxygen saturation, arterial blood pressure, temperature and urine output, throughout the procedure. These monitoring modalities are crucial for detecting acute hemodynamic or electrophysiological changes that may occur suddenly during cardiac catheterization. Additionally, regional cerebral oxygenation was monitored using near-infrared spectroscopy (NIRS) in all neonatal patients, which is of particular importance in this vulnerable population given their limited cerebral autoregulatory capacity. For high-risk cases, advanced life support equipment—including extracorporeal membrane oxygenation (ECMO) devices—along with necessary vasoactive inotropic drugs and infusion pumps were kept readily available in the operating room, thereby ensuring immediate intervention in the event of a critical deterioration. To prevent contrast-related allergic reactions, all patients received premedication with diphenhydramine and dexamethasone after intravenous access was secured, which reflects current best-practice protocols. Supplemental oxygen was administered via face mask at a rate of 2–4 L/min, with continuous monitoring of oxygen delivery effectiveness. Furthermore, all patients were actively warmed using thermal devices to prevent procedure-related hypothermia, as temperature instability has been shown to increase perioperative morbidity in pediatric populations. This comprehensive strategy highlights the importance of a multidisciplinary and precautionary approach to peri-procedural care in children undergoing cardiac catheterization.

General anesthesia was administered to patients undergoing interventional procedures such as pulmonary valve replacement, patent ductus arteriosus (PDA) stenting, and atrial or ventricular septal defect (ASD/VSD) closure, where immobility and optimal procedural conditions are essential. Anesthesia induction was achieved with a combination of hypnotic agents—midazolam (0.1 mg/kg), ketamine (1–2 mg/kg), propofol (0.5–3 mg/kg)—together with the neuromuscular blocker rocuronium (0.6 mg/kg) to facilitate airway control and secure hemodynamic stability. Maintenance of anesthesia was achieved with 1–2% sevoflurane, chosen for its favorable hemodynamic profile and rapid recovery characteristics in pediatric patients. All anesthetic agents were carefully titrated based on patient age, weight, and underlying cardiac physiology to minimize cardiovascular depression. For diagnostic procedures not requiring deep anesthesia, sedoanalgesia was preferred, thereby minimizing drug exposure and potential adverse events. Sedoanalgesia was induced with bolus or infusion of midazolam, ketamine, propofol, or remifentanil (0.05 mcg/kg/min), adjusted according to the patient’s clinical condition and procedural requirements. Continuous monitoring allowed titration to achieve the desired depth of sedation while preserving spontaneous respiration whenever possible. Sedation levels for all procedures were objectively assessed using the Ramsay Sedation Score (RSS) [7], which provided a standardized and reproducible measure of patient responsiveness, ensuring both patient safety and procedural efficacy. The anesthesiology team maintained close collaboration with interventional cardiologists throughout each procedure to optimize patient outcomes. This structured approach highlights the balance between adequate sedation, rapid recovery, and minimization of anesthesia-related complications in pediatric cardiac catheterization.

1 Anxious, agitated or restless.
2 Cooperative, oriented and calm.
3 Responds to commands only.
4 Brisk response to light glabellar tap or loud auditory stimulus.
5 Sluggish response to light glabellar tap or loud auditory stimulus.
6 No response to stimulus.

If the targeted sedation level was not achieved, airway safety was ensured with orotracheal intubation or a laryngeal mask.

### 2.2. Catheterization Risk Score for Pediatrics (CRISP)

The CRISP is an 11-component scoring system developed through expert consensus, with a total score range of 0–21. It includes patient status/timing of catheterization (X1), age (X2), weight (X3), inotropic support (X4), respiratory status (X5), systemic illness/failure (X6), ASA score (X7), physiological category (X8), pre-catheterization diagnosis (X9), procedure risk category (X10), and Procedure Type (X11) [6].

For this study, we applied the CRISP classification proposed by Nykanen et al., which divides patients into five separate SAE risk categories based on CRISP scores. The final CRISP scores and point allocations for each variable were calculated and entered into the study database [6].

### 2.3. Definition of Serious Adverse Events (SAE)

Serious adverse events were defined according to the Congenital Cardiovascular Interventional Study Consortium (CCISC) registry [6]. An SAE was defined as any adverse event causing death, permanent morbidity, need for additional intervention, or prolonged hospital stay.

Deaths, permanent arrhythmias, bleeding requiring blood transfusion, desaturation or respiratory arrest, hypotension requiring inotropic support, cardiac perforation, stent embolization, and complications requiring emergency surgery were classified as major complications, as these events are associated with significant morbidity and often necessitate advanced therapeutic interventions. In particular, life-threatening arrhythmias such as complete atrioventricular block, ventricular tachycardia, or ventricular fibrillation may require immediate pacing, antiarrhythmic medication, or resuscitative efforts. Similarly, complications such as cardiac perforation or device embolization typically mandate urgent surgical or interventional retrieval, thereby increasing the procedural risk profile. In contrast, non–life-threatening events such as vascular injury, transient arrhythmias, hypotension not requiring inotropes, laryngospasm, and allergic reactions were classified as minor complications, since these conditions are generally reversible with conservative measures and do not usually prolong hospitalization. Nevertheless, even minor events can impact patient recovery, resource utilization, and family counseling, highlighting the importance of systematic complication monitoring. Deaths occurring within the first 24 h after completion of cardiac catheterization and angiography were considered procedure-related mortality, reflecting international consensus definitions and ensuring comparability across studies. This classification framework provides a standardized approach to reporting adverse outcomes and facilitates benchmarking of safety and quality in pediatric cardiac catheterization.

## 3. Statistical Analysis

Statistical analyses were conducted using IBM SPSS version 22.0 (IBM Corp., Armonk, NY, USA). Descriptive data were presented as median (interquartile range [IQR]) or as counts and percentages. Normality of data distribution was assessed using the Kolmogorov–Smirnov test. Chi-square and Mann– Whitney U tests were used for the comparison of groups, A receiver operating characteristic (ROC) curve was generated to evaluate the performance of CRISP in our population. Chi-square test was used to assess the predictive ability for SAEs. A *p*-value of <0.05 was considered statistically significant.

## 4. Results

After applying the exclusion criteria, a total of 700 patients were included in this study. Among the cases, 16% (*n* = 112) were neonates, and 58% had single-ventricle physiology. Interventional procedures were performed in 72% of the patients. The median CRISP score was 8 (IQR 6–10). Patient and procedural characteristics are summarized in Table 1.

A total of 54 serious adverse events (SAEs) were identified, representing an incidence of 7.7%. The most commonly observed SAEs were arrhythmias requiring pharmacologic intervention (26%), airway compromise (14.8%), increased need for hemodynamic support (11%), and pulmonary compromise (11%). Two patients died during the procedure. The types and distribution of SAEs are shown in Table 2.

The incidence of SAEs was analyzed according to CRISP score categories. SAE rates in patients with CRISP Categories 1 through 5 were 2.9%, 4.3%, 11%, 17.3%, and 41%, respectively (Table 3). The incidence of SAEs increased with higher CRISP scores and categories. The area under the ROC curve was 0.84 (95% Confidence Interval: 0.76–0.92; *p* < 0.05), indicating good predictive ability of the CRISP score for SAEs. The incidence of major complications was higher in the High CRISP Risk Category.

## 5. Discussion

In this study, we investigated the predictive value of the CRISP in estimating the occurrence of SAEs in pediatric patients undergoing cardiac catheterization and angiography at a high-volume cardiac center. Our findings showed that SAEs occurred in 7.7% of procedures, and the likelihood of SAEs increased with higher CRISP scores and categories. This study represents one of the relatively few investigations in the literature evaluating this aspect in pediatric cases. At the same time, it has been observed that the CRISP score can be used in a population with a high prevalence of single-ventricle physiology and a high rate of interventional procedures.

Recent technological advancements have enabled a broad range of diagnostic and interventional procedures in pediatric patients with various cardiac conditions. Accordingly, due to the development of non-invasive imaging modalities such as echocardiography, computed tomography (CT), and magnetic resonance angiography (MRA), the proportion of interventional cardiac catheterization and angiography procedures has been increasing. In fact, compared with previously reported interventional rates of 30% by Tokel et al. [8] and 50% by Shim et al. [9], the interventional rate in our study was higher, at 72%.

The incidence of complications in pediatric cardiac catheterization and angiography is influenced by both the patient’s diagnosis and the nature of the procedure. Several demographic and clinical factors—such as age, weight, clinical condition at the time of intervention, underlying disease, diagnostic versus interventional intent of the procedure, anesthetic management, and the cardiologist’s skill and experience—affect the risk of complications [10,11,12]. These complications can range from minor events to life-threatening situations requiring emergency open-heart surgery, permanent sequelae, or even death. Reported complication rates in pediatric patients vary widely in the literature, ranging from 2% to 40%. Şahin et al. [3] reported an SAE incidence of 9.1%, and Gerçeker et al. [13] reported 10.7%. The incidence in our study was 7.7%, which is comparable with the existing literature.

Pediatric cardiac catheterization is a complex procedure with increased risks, particularly in younger patients and those with low body weight. One of the major contributing factors to the increased complication rate in such patients is hemodynamic instability due to their immature physiological systems. A standardized preoperative assessment tool is essential for predicting serious adverse events in pediatric cardiac catheterization. In 2015, the CCISC introduced the CRISP scoring system. This validated tool consists of 21 questions and is designed to estimate the likelihood of procedure-related SAEs. By applying the CRISP score, healthcare providers can better prepare equipment, staff, and supportive resources in advance to mitigate SAE risk [6,12].

The CRISP score has been proven to be a reliable predictor of SAEs, with an area under the curve (AUC) of 0.71 (95% CI: 0.66–0.91; *p* < 0.008) demonstrating good discriminative ability [14]. Another multivariate analysis further supported its predictive value with an AUC of 0.741 [6]. In our study, the CRISP score also showed strong predictive accuracy, with an AUC of 0.84 (95% Confidence Interval: 0.76–0.92; *p* < 0.05), indicating excellent discriminative performance.

Risk stratification using CRISP in cardiac catheterization and angiography cases supports its association with SAE incidence. Nykanen et al. [6] divided CRISP scores into five distinct categories with corresponding SAE rates of 1.0%, 2.6%, 6.2%, 14.4%, and 36.8%, respectively, facilitating clinical decision-making. In another study by Santos et al., the incidence of SAEs by CRISP category was reported as 7.3%, 12.2%, 36.6%, and 34.2%. In our study, the incidence of SAEs across CRISP Categories 1 through 5 was 2.9%, 4.3%, 11%, 17.3%, and 41%, respectively.

## 6. Limitations

The primary limitation of this study is its retrospective and single-center design. Retrospective data collection may introduce selection bias and limit the ability to capture all relevant clinical variables that could influence outcomes. Another limitation is that operator-dependent variables were not evaluated, as the procedures were performed by three different teams. The lack of inter-operator variability analysis may mask important differences in technique, experience levels, and decision-making processes that could affect complication rates. The higher proportion of interventional procedures compared to diagnostic procedures may have influenced the rate of serious adverse events. Additionally, our patient population had a high prevalence of single-ventricle physiology, which may limit the generalizability of our findings to centers with different case mixes. The absence of long-term follow-up data prevents assessment of delayed complications or outcomes that may manifest beyond the immediate periprocedural period. Furthermore, the study period coincided with the implementation of new protocols and equipment, which may have introduced temporal confounding variables. Finally, the relatively small sample size in higher CRISP risk categories may limit the statistical power to detect significant differences in these subgroups.

## 7. Conclusions

CRISP demonstrated good predictive value in this single-center cohort and may be a useful tool for risk stratification and can predict the development of serious adverse events. Our findings confirm that CRISP provides excellent discriminative ability with an area under the curve of 0.84, which compares favorably to previously published validation studies. The progressive increase in SAE rates across CRISP categories (from 2.9% to 41%) demonstrates the score’s clinical utility in identifying high-risk patients who require enhanced monitoring and resource allocation.

Therefore, its implementation may have a positive impact on patient care by supporting better planning of pre- and post-catheterization management. The ability to predict procedural risk before intervention allows for proactive measures including appropriate staffing levels, availability of specialized equipment such as ECMO, and informed consent discussions with families. High-risk patients can be scheduled during optimal staffing periods with experienced teams and immediate surgical backup availability.

Beyond individual patient management, CRISP can serve as a valuable benchmarking tool for quality improvement initiatives and inter-institutional comparisons. The standardized risk assessment facilitates meaningful analysis of complication rates adjusted for case complexity, enabling centers to identify areas for improvement and track performance over time. Furthermore, the score can guide resource allocation decisions and help justify the need for specialized equipment and personnel in pediatric cardiac catheterization laboratories.

Future research should focus on validating CRISP in diverse populations, investigating its utility in guiding specific interventions for high-risk patients, and exploring the potential for real-time risk modification strategies. The integration of CRISP into electronic health records and clinical decision support systems could further enhance its clinical utility and ensure consistent application across different institutions.

## Figures and Tables

**Table 1:** Patient and Procedure Characteristics.

Variables	*n* = 700
Newborn	112 (16)
Age < 1 year	238 (34)
Male	350 (50)
Weight < 5 kg	168 (24)
Accompanying syndromes (Down, Turner, Williams, Eisenmenger)	49 (7)
Single ventricle	406 (58)
Procedure duration	40 (30–55)
Urgent procedure	77 (11)
Procedure type	
Diagnostic procedure	196 (28)
Interventional procedure	504 (72)
Anesthesia management	
Sedoanalgesia	476 (68)
Laryngeal mask airway	140 (20)
Endotracheal tube	84(12)
ASA class	
II	168 (24)
III	322 (46)
IV	210 (30)
CRISP score	8 (6–10)
Serious Adverse Event	54 (7.7)

*n* (%) or median (interquartile range) ASA: The American Society of Anesthesiologists, CRISP: Catheterization Risk Score for Pediatrics.

**Table 2:** Serious Adverse Events.

Type of Complications	
**Major complications**	34 (63)
Permanent or fatal arrhythmia	5 (9.2)
Complete atrioventricular block	1 (1.8)
Ventricular tachycardia	3 (5.5)
Ventricular fibrillation	1 (1.8)
Increase in hemodynamic support	6 (11)
Cardiac arrest	5 (9.2)
Airway compromise, unanticipated	4 (7.4)
Death	2 (3.7)
Device migration requiring open surgical removal, removal via cut down, or transcatheter retrieval	2 (3.7)
Pericardial effusion requiring surgical intervention or pericardial drainage	2 (3.7)
Pulmonary compromise (pulmonary hemorrhage/hemoptysis or pulmonary edema)	6 (11)
Retroperitoneal hematoma	2 (3.7)
**Minor complications**	20 (37)
Transient arrhythmia	9 (16.6)
Complete atrioventricular block	3 (5.5)
Supraventricular tachycardia	6 (11)
Allergic reactions	4 (7.4)
Vascular injury	3 (5.5)
Laryngospasm	4 (7.4)
Total	54 (100)

**Table 3:** Serious Adverse Events (SAEs) According to the CRISP Risk Category.

CRISP Score	Risk Category	All Patients *n*	Patients Having Complication SAE (*n* = 54) *n*	Major Complication (*n* = 34) *n*	Minor Complication (*n* = 20) *n*	Patients Having Complication SAE %	Expected SAE (%)
0 to 2	CRISP 1	93	2	1	1	2.9	1
3 to 5	CRISP 2	348	15	8	7	4.3	2.6
6 to 9	CRISP 3	190	21	12	9	11	6.2
10 to 14	CRISP 4	52	9	7	2	17.3	14.4
15 or more	CRISP 5	17	7	6	1	41	36.8

## Data Availability

The data that support the findings of this study are available from the corresponding author upon reasonable request. Further inquiries can be directed to the corresponding author.

## Abbreviations

SAE	Serious adverse event
CRISP	Catheterization Risk Score for Pediatrics
